# Supplementary material for: Investigator-initiated versus industry-sponsored trials – visibility and relevance of randomized controlled trials in clinical practice guidelines (IMPACT)
Source: BMC Med Res Methodol. 2025 Mar 27;25:80. doi: 10.1186/s12874-025-02535-z (PMC11948659; doi:10.1186/s12874-025-02535-z)
Supplement: Supplementary file 1 — Additional file 1. Illustration of the process of identifiying forward citations of RCTs, adapted from Nury et al. [4]. [file 12874_2025_2535_MOESM1_ESM.pdf]

Additional file 1

(Hecht et al. Investigator-initiated versus industry-sponsored trials – Visibility and relevance of randomized controlled trials in clinical practice guidelines (IMPACT))

Illustration of the process of identifiying forward citations of RCTs

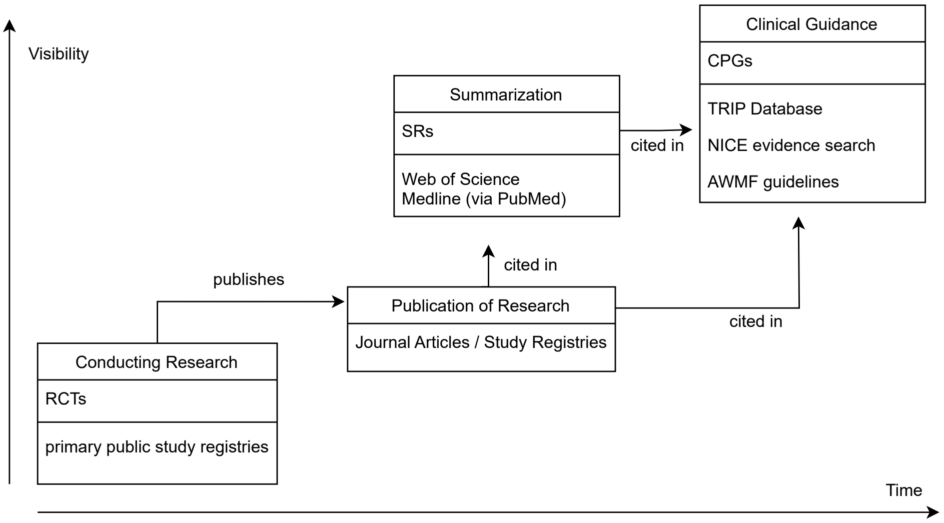

Fig. 1: Process of identifiying forward citations of RCTs. This illustration is adapted from Nury et al. [1].

References

[1] Nury E, Bischoff K, Wollmann K, Nitschke K, Lohner S, Schumacher M, et al. Impact of investigator initiated trials and industry sponsored trials on medical practice (IMPACT): rationale and study design. BMC Medical Research Methodology. 2020 Dec;20(1):246. https://doi.org/10.1186/s12874-020-01125-5.
