# Supplementary material for: Investigator-initiated versus industry-sponsored trials – visibility and relevance of randomized controlled trials in clinical practice guidelines (IMPACT)
Source: BMC Med Res Methodol. 2025 Mar 27;25:80. doi: 10.1186/s12874-025-02535-z (PMC11948659; doi:10.1186/s12874-025-02535-z)
Supplement: Supplementary file 3 — Additional file 3. Results from additional regression models performed as sensitivity analyses with log10 transformed sample size (instead of dichotomized sample size). [file 12874_2025_2535_MOESM3_ESM.pdf]

Additional file 3

(Hecht et al. Investigator-initiated versus industry-sponsored trials – Visibility and relevance of randomized controlled trials in clinical practice guidelines (IMPACT))

Results from additional regression models performed as sensitivity analyses with log10 transformed sample size (instead of dichotomized sample size)

Table 1: Multivariable Negative-Binomial-Regression-Models for a) Impact-on-CPGs and b) Impacted-CPGs by trial. The observed time per trial was taken into account as an offset term after log-transformation

	**Impact-on-CPGs per trial**			**Impacted-CPGs per trial**		
**Covariates**	IRR	95% CI	p-value	IRR	95% CI	p-value
**Cohort**						
German IITs	-	-	-	-	-	-
International IITs	1.47	0.66,3.01	0.3	1.58	0.89,2.82	0.12
German ISTs	1.31	0.58,2.95	0.5	1.47	0.80,2.68	0.2
International ISTs	0.44	0.20,0.92	0.033	0.64	0.35,1.14	0.14
**Drug trial**						
no	-	-	-	-	-	-
yes	0.81	0.49,1.35	0.4	1.03	0.70,1.51	0.9
**log10(Study size)**	8.09	4.24,15.9		4.52	2.83,7.37	
			< 0.001			< 0.001
**Number of primary outcomes**						
> 1	-	-	-	-	-	-
1	0.49	0.26,0.87	0.015	0.63	0.40,0.97	0.041

IRR = Incidence Rate Ratio, CI = Confidence Interval

Table 2: Multivariable Cox-Proportional-Hazard-Model for the time from Study-Start to Guideline-Impact. For definition of event and time-to-event see also description of Figure 5.

**Covariates**	**HR**	**95% CI**	**p-value**
**Cohort**			
German IITs	-	-	-
International IITs	1.31	0.83,2.05	0.2
German ISTs	1.05	0.65,1.68	0.9
International ISTs	0.61	0.37,1.01	0.055
**Drug trial**			
no	-	-	-
yes	1.00	0.72,1.39	> 0.9
**log10(Study size)**	3.03	2.10,4.35	< 0.001
**Number of primary outcomes**			
> 1	-	-	-
1	0.94	0.64,1.37	0.7

HR = Hazard Ratio, CI = Confidence Interval
